# BLUEPRINT TO TARGET THE SPINAL CORD MOTOR CIRCUITRY TO PRESERVE AND RESTORE MOBILITY IN OLD AGE

**DOI:** 10.1093/geroni/igad104.0730

**Published:** 2023-12-21

**Authors:** Gregorio Valdez

**Affiliations:** Brown University, Providence, Rhode Island, United States

## Abstract

Spinal motor neurons are central for the initiation and modulation of all voluntary movements. Within the spinal cord, motor neurons form tens of thousands of excitatory (glutamatergic and cholinergic) and inhibitory (GABAergic and glycinergic) synapses along their dendritic arbor and soma. These synapses contain the information required to execute fine and complex motor commands and are together referred to as the motor circuitry. Once activated, motor neurons drive muscle contraction by releasing neurotransmitters from their axon terminals at neuromuscular junctions (NMJs). Thus, either the death of motor neurons or their disconnection from other neurons are skeletal muscles during aging would undoubtedly compromise motor function. We will show that motor neurons do not die in old female and male mice, rhesus monkeys, and humans. Instead, these neurons selectively and progressively shed excitatory synaptic inputs throughout the soma and dendritic arbor during aging. Thus, aged motor neurons contain a motor circuitry with a reduced ratio of excitatory to inhibitory synapses that may be responsible for the diminished ability to activate motor neurons to commence movements. Additionally, we will show that aged motor neurons present with aberrant changes in genes and molecular pathways with roles in glia-mediated synaptic pruning, inflammation, axonal regeneration, and oxidative stress. I will bring these findings together to suggest that motor neurons at a minimum contribute to the loss of their own synapses with advancing age and discuss possible avenues for therapeutic intervention.

